# 

**Published:** 1898-02

**Authors:** 


### INDEX TO VOLUME XIV.

ORIGINAL COMMUNICATIONS—

Abscess (Tropical) of Liver (Jackson) .......... 433
Acne, Treatment of (Gottheil) .......... 77
Anemia and its Treatment (Felder) .......... 95
Anesthesia, Major, General Considerations upon (Dawbarn) .......... 361
Anesthesia, Threatened Death during (Dawbarn) .......... 439, 505
Cancer of the Uterus (Goggans) .......... 73
Concussion of Brain (Clark) .......... 448
Chloroform in Labor, Therapeutic Application of (Upshur) .......... 89
Consumption, Cause and Prevention of (Todd) .......... 629
Convergent Squint, Tenotomy in (Patterson) .......... 665
Cystotomy (suprapubic) for Stone (Harrison) .......... 525
Development of the Human Race and Obstetric Nursing (Kime)... 8, 83, 389
Diastase in Therapeutics (Fite) .......... 24
Diphtheria Antitoxin (Slack) .......... 577
Diphtheria, Laryngeal, treated by Antitoxin and Calomel Fumigation (Levy) .......... 319
Diseases of Ear, Nervous Troubles Associated with (Foucher) .......... 597
Drinking-Water, Value of (Kilpatrick) .......... 658
Effect of Intense Flashes of Electric Light upon the Eye (Roy) .......... 802
Entero-Colitis of Infancy (Clark) .......... 222
Expert Testimony in Criminal Cases Involving the Plea of Insanity (Olmsted) .......... 217
Experience Gleaned from Eye and Ear Practice (Ellett) .......... 747
Fistula in Ano, Remarks on (Goldsmith) .......... 314
Forceps Rotation in Posterior Positions of Vertex (Gillespie) .......... 519
Gall-Bladder and Ducts, Surgery of (Gaston) .......... 694, 729
Gunshot Wounds, Two Cases of,—Axilla and Popliteal Space,—Ligations—Secondary Amputation (Hudson) .......... 4
Hysteria in Early Life (Eshner) .......... 603
Importance of High Grade of Physical Health for Dependent and Delinquent Classes in Public Institutions (Spratling) .......... 721
Infant Mortality, Its Prime Cause and Prevention (Van Goidtsnoven) 303
Inguinal Adenitis, Abortive Treatment of Acute Suppurative, by Pressure Bandage (Slack) .......... 239
Iron, Administration of (Felder) .......... 667
Kidney, Gunshot Wound of (Hudson) .......... 515
Lactophenin (Childs) .......... 175
Lingual Tonsil, Hypertrophy of (Adams) .......... 237
Malignant Growths, Blue Pyoktanin in the Treatment of (Slack) .......... 377

ORIGINAL COMMUNICATIONS—*continued.*
Malignant Tumor, Two Cases of, Affecting the Breast, and their Treatment (Hutchins) ........ 369
Nephrectomy for Large Multiple Abscess of the Kidney (Duggan)...... 93
New Form of Treatment in Uterine Disease (Marcus) ........ 818
Nineteenth Century Medicine (Battey) ........ 152
Obstetric Surgery, Clinical Notes (Allen) ........289, 588
Otitis Media, Non-Suppurative (Smith) ........ 584
President's Address Medical Association of Ga., 1897 (Noble)........ 145
Psychiatry in Southern States (Powell) ........ 736
Puerperal Infection (Graham) ........ 815
Quinine in Malaria, Excluding the Simple Intermittents (Van Marter)... 793
Rhinoplastic Operation for Loss of Nose (Crawford)........ 384
Sebaceous Tumors and their Cure (Patterson) ........ 94
Skin and Venereal Diseases. Some Interesting Diseases of (Wolff)........ 19
Some Surgical Operations Recently Performed in the Macon Hospital (Williams) ........ 160
Spray for Nose and Throat (Brown) ........ 759
Transfusion, A Simple Method of (Cooper)........ 1
Treatment of Gonorrhea by Hydrostatic Irrigation (Champion) ........ 810
Trigeminal Paresthesia (Pope) ........ 452
Urethral Diseases, Meddlesome Instrumentation in (Champion) ........ 230
Warts and their Treatment (Hutchins) ........ 663

SOCIETY REPORTS—
Atlanta Society of Medicine........397, 323, 98, 458, 612
Clinical Society of St. James Dispensary ........ 821
Medical Association of Georgia........178
Richmond Academy of Medical Surgery........30, 678
Tri-State Medical Society, Alabama, Georgia and Tennessee........669

CORRESPONDENCE—
A Bill to Regulate the Employment of and Pay for Expert Medical Testimony (Terrell)........ 329
Acute Tonsillitis (Foy)........ 331
American Medicinal Flora........473
Antitoxin in Membranous Croup (Barnum) ........532
Card from Dr. J. F. Alexander........ 194
Criminal Abortion (Arnold) ........ 829
Efficient Vaccination (Stirling)........ 682
Medical Expert—What he is and what he should be (Hagan) ........529
New York Letters (Ludlow)........39, 106, 761
Notes from London (Campbell) ........327, 470
Notes upon a Visit to New York and Baltimore (Davis) ........ 246
Ovarian Cysts in Negresses (Stone)........536
Some Needed Legislation (Bizzell) ........ 408
The Board of Censors, Medical Ethics, Newspapers, etc. ("Code")........ 241
The Plague in India (Mitra) ........ 402
Venomous Snakes (Stirling)........534

EDITORIAL—

Announcement .... 773
College Commencements.... 113
Defective Medical Education.... 769
Dr. Crawford W. Long and Anesthesia.... 540
Ethics and the Newspapers.... 250
Examinations by State Boards, Atlanta, June 29, '97.... 413
Football .... 683
Football, National Quarantine, and Governor's Veto.... 771
Jubilee Meeting of American Medical Association .... 332
Medical Association of Georgia.... 112
Medical Expert Testimony—Abuse and Remedy.... 616
Medical Matters in 1897.... 831
Mob Law and its Remedy.... 477
Notice to Subscribers .... 543
Osteopathy—What is it? .... 412
Patented Foreign and Proprietary Domestic Preparations.... 48
Southern Section Laryngological, Rhinological and Otological Association 773
Southern Surgical and Gynecological Association.... 685
Successful Esophago-Enterostomy.... 834
The Antivivisection Movement .... 45
The Board of Health and Dr. Olmsted's Bill .... 836
The Committee on Legislation.... 411
The Medical Association's Meeting at Macon.... 195
Tri-State Medical Society, Alabama, Georgia and Tennessee.... 541
Vivisection in the District of Columbia.... 335
Will it Come to This? .... 109
Yellow Fever and its Bacillus.... 537

SELECTIONS AND ABSTRACTS—

Exception to "Colles's Law," 481; Fatty Seborrhea and Alopecia Areata, 482; Treatment of Cutaneous Cancer, 482; Carcinoma of Bones, 483; Treatment of Pruritus, 483; Treatment of Sarcoma by Electrolysis and Cataphoresis, with Internal use of Donovan's Solution, 484; Treatment of Eczema by Picric Acid, 485; Varicellous Staphylococcia, 486; Contribution to Therapeutics of Iron, 486; Specific Action of Quinine in Malaria, 492; Aristol in Operation on Nose, 494; Instruction in Administration of Anesthetics, 494; Physicians in United Sates, 495; Physicians on Witness stand, 544; Test of Responsibility of Insane, 551; Foreign Literature on Appendicitis, 555; Chronic Gastritis, 557; Action of Scopolamine on Iris and Ciliary Muscle, 559; Antiseptic and Climatic Treatment of Pulmonary Tuberculosis, 561; Apenta Water, 565; Southern States Health Record, 565; First Cesarean Section in Georgia, 567; Study of Pyemia and Sepsis, 567; Specialists and Specialities, 568; Gynecological Gleanings, 569-634; Hematuria, Its Relation to Malarial Infection and Ingestion of Quinine, 611; Cost of Yellow Fever, 615; Treatment of Yellow Fever, 621; Opening Mastoid Cells, 623; Surgical Treatment of of Fibroid Tumors of Uterus, 625; Use of Opium in Diarrheal Diseases of

SELECTIONS AND ABSTRACTS—*continued.*

Children, 626; Clinical Cases under Pepto-Mangan, 627; Homatropine in Refraction, 628; Defective Eyesight in Children, 629; Increase of Insanity and Consumption among Negroes since the War, 631; Holocaine, 633; Negro and Locomotor Ataxia, 634; Gargle for Follicular Tonsillitis, 634; Boils, 635; Rejections for Life Insurance, 635; Decadence of Syphilis, 636; Otorrhea and Life Insurance, 688; Formalin in Ophthalmology, 690; Experiments on Union of Corneal Wounds, 691; Treatment of Styes, 692; Treatment of Ophthalmia Neonatorum, 693; Treatment of Pterygium, 694; Ichthyol in Ophthalmology, 694; Hydrozone for Diseases of the Genito-Urinary Tract, 694; Surgical Attributes of Negro, 696; National Health Board, 698; Woodbridge Treatment of Typhoid Fever, 702; Compensation of Medical Witnesses, 703; Widal Test for Typhoid Fever, 703; Treatment of Acute Tonsillitis, 704; Pleurisy with Effusion, 706; Alcoholic Mania, 706; Administration of Cod-Liver Oil, 707; When Doctors Disagree, 712; Vomiting in Pregnancy, 768; How to Cook a 'Possum, 787; Some Lessons of the Yellow Fever Epidemic, 788; Typho-Malarial Fever becoming Obsolete, 789; Football, 790; For Alcoholic Neurasthenia, 790; Formaldehyde Gas, 791; Salve for Hemorrhoids, 792; For Neurasthenia, 792; Case of Pyemia after Acute Suppuration of the Ear, 417; Ocular Disease from Gout, 418; Hysteria with Strabismus and Ptosis, 419; Osteomata of the Auditory Canal, and their Treatment, 420; Enucleation and Exenteration, 421; How Long is Syphilis Contagious, 451; The Technique of Vaginal Section Irrespective of Hysterectomy, for Diseased Appendages and Small Pelvic Tumors, 434; Hand Disinfection, 425; Treatment of Fractures, 426; Kryofin, 426,, A Minister on Faith Cure and Christian Science, 336; The Local Treatment; of Chronic Gastric Catarrh, 337; Ferratin, 340; Continued Malarial Fever, A Medical Myth, 342; Heredity—Its Significance in Life Insurance, 345; Reciprocity in Medical Licensure, 348; The American Pediatric Society's Report on the Collective Investigation of the Antitoxin Treatment of Laryngeal Diphtheria in Private Practice, 1896–1897, 255; When to call a Surgeon in Appendicitis, 259; Report on Malarial Hematuria, 263; The Treatment of Cancer, 268; Some Important Facts About Chloroform, 270; Notes on the Treatment of Anemias, 273; The Removal of Cerumen from the External Auditory Canal, 275; Retroversion of the Uterus, 277; Early Signs of Locomotor Ataxia, 279; The Janet Abortive Treatment for Gonorrhea, 281; How Far Can the Surgeon Go? 282; Three Obstetrical Warnings, 283; Trachoma Among Negroes, 283; Acute Follicular Tonsillitis, 283; Test for Sugar in the Urine, 168; Spurs of the Nasal Septum as Factors in Diseases of the Respiratory Tract, 198; Cleanliness in Catarrh, 199; Eucaine in Laryngology and Rhinology, 200; Treatment of Trachoma, 201; The Furor Scribendi, 202; A New Method of Dealing with the External Meatus in Operations upon the Mastoid, 203; Foreign Bodies in the Ear, 204; Results of Gynecological and Abdominal Surgery, 205; How Chloroform Kills, 206; Fistula in Ano, 207; Ten-Cent Journalism, 207; Some Supplementary Remarks on Scopolamine as a Mydriatic, 208; To Find the Gonococcus, 208; The Prevention of Iodism in the Use of Potassium Iodide, 209; Causes

SELECTIONS AND ABSTRACTS—*continued.*

of Glaucoma, 116; Scopolamine in Plastic Iritis, 117; Foreign Bodies in the Ear, 118; Prevention of Ophthalmia Neonatorum, 119; Operation for Mastoiditis, Complicated by Hemorrhage from the Lateral Sinus—Recovery, 120; Headaches from Eye-Strain, 120; Recent Advances in Rectal Surgery, 121; Notes on the Treatment of Fecal Fistulæ, 124; The Civil Liability of Physicians and Surgeons in Cases of Malpractice, 127; The Status of the Antitoxin, 129; Cocaine Anesthesia, 131; Early Diagnosis of Carcinoma of the Breast, 132; Southern Georgia as a Winter Resort, 134; Vaginal Ligation of the Uterine Arteries for Uterine Fibromata, 135; Laparotomy for Perforating Typhoid Ulcers, 136; Mortality Among Negroes, 137; Semi-Centennial of the American Medical Association, 38; Surgery of Empyema, 44; The Passing of the Reflex, 55; The Confessions of a Cocainist, 60; Irregular Menstruation in Young Women due to Anemic Conditions, 852; The Value of Potassium Permanganate as an Antidote for Morphine, 854; Port Mortem Delivery, 855; A National Quarantine Law, 856; The National Quarantine Bill, 857; For Removal of Warts and Corns, 858; When shall we use the Forceps, 858; Requirements of the Various States Regarding the License to Practice Medicine, 859; Baby Incubation in New York, 859; Suprapubic Cystotomy, 860; Hysteria Due to Pelvic Disease, 861; Is Crime a Disease? 861; Zinc Permanganate in Gonorrhea, 862; Indication for the Induction of Abortion, 862; Agreeable Salicylate Mixture, 864.

MEDICAL ITEMS..........62, 139, 210, 284, 350, 428, 496, 570, 637, 708, 774, 838

BOOK REVIEWS—

*About Children.* (Kelley)........ 784
*American Year Book of Medicine and Surgery* (Gould)........ 71
*Anomalies and Curiosities of Medicine* (Gould.)........ 69
*A Practical Treatise on Diseases of the Skin.* (Hyde)........ 288
*Clinical Manual.* (MacFarland)........ 647
*Clinical Methods.* (Hutchinson and Rainey.)........ 849
*Clinical Text-Book of Surgical Diagnosis and Treatment.* (McDonald.)...... 848
*Constipation in Adults and Children.* (Illoway)........ 785
*Cutaneous Medicine, Treatise on Diseases of Skin.* (Duhring)........ 715
*Deutsch's Letters.* (Deutsch)........ 71
*Diseases of the Ear, Nose and Throat* (Bishop)........ 286
*Diseases of the Eye.* (Nettleship.)........ 782
*Diseases of Women.* (Sutton and Gibbs)........ 643
*Elementary Bandaging and Surgical Dressing.* (Pye)........ 144
*Elements of Latin.* (Crothers and Bice.)........ 850
*Elementary Principles of Electro-Therapeutics.* (Haynes)........ 432
*Epitome of History of Medicine.* (Park)........ 714
*Essays on Social Topics.* (Cook)........ 504
*Eye-Strain in Health and Disease.* (Ranney)........ 575
*Flint's Medical and Surgical Directory of U. S. and Canada*........ 359
*Fracture of the Radius.* (Roberts)........ 431
*High Altitudes for Consumptives.* (Tussey)........ 70

BOOK REVIEWS—*continued.*

*Hypnotism.* (Wetterstrand)........ 644
*Hysteria and Certain Allied Conditions.* (Preston)........ 432
*Illustrated Skin Diseases.* (Gottheil)........ 65, 360
*Illustrated Skin Diseases, Atlas and Text-Book.* (Gottheil.)........ 850
*Incompatibilities in Prescriptions.* (Ruddiman)........ 718
*Inebriety.* (Palmer)........ 144
*International Medical Annual.* (Treat)........ 144
*Lectures on Action of Medicines.* (Brunton)........ 646
*Lectures on Malarial Fevers.* (Thayer)........ 783
*Lectures on Renal and Urinary Diseases.* (Saundby)........ 214
*Leprosy and the Charity of the Church.* (Mulhane)........ 288
*Lippincotts' Magazine for January,* 1898........ 857
*Liver of Dyspeptics.* (Boix)........ 646
*Living Age for* 1898........ 720
*Living Substance as Such and as Organism.* (Andrews)........ 781
*Lofoten Islands*........ 786
*Manual of Medical Jurisprudence.* (Taylor)........ 643
*Manual of the Practice of Medicine.* (Stevens)........ 143
*Manual of Static Electricity in X-ray and Therapeutic uses.* (Morell)........ 431
*Medical News Visiting List for* 1898........ 719
*Outlines of Rural Hygiene.* (Bashore.)........ 849
*Pathological Technique.* (Mallory and Wright.)........ 848
*Pocket Medical Dictionary.* (Warner)........ 216
*Practical Treatise on Diseases of the Skin.* (Shoemaker.)........ 780
*Practical Treatise on Sexual Disorders, Male and Female.* (Taylor)........ 645
*Principles of Medicine.* (Mack)........ 576
*Practical Treatise on Materia Medica and Therapeutics.* (Bartholow)........ 648
*Reference Book of Practical Therapeutics.* (Foster)........ 503
*Retinoscopy.* (Thorington)........ 287
*Surgery of Rectum and Pelvis.* (Kelsey)........ 503
*Surgical Hints for the General Practitioner.* (Lilienthal)........ 360
*System of Diseases of Eye.* (Norris and Oliver)........ 357, 499
*System of Medicine.* (Allbutt)........ 358, 713, 846
*System of Practical Medicine.* (Loomis and Thompson)........ 501
*Text Book of Diseases of Women.* (Penrose)........ 502
*Text Book of Practical Therapeutics.* (Hare)........ 716
*Text-Book of Practice of Medicine.* (Anders)........ 717
*Text Book for Nurses.* (Wise)........ 783
*The Diseases of Infancy and Childhood.* (Holt)........ 68
*The Diseases of the Stomach.* (Ewald)........ 215
*The Medical Environment.* (Black)........ 72
*Tonic Treatment of Syphilis.* (Keyes)........ 719
*Traumatic Injuries of the Brain and its Membranes.* (Phelps.)........ 847

### CONTRIBUTORS TO VOLUME XIV.

Adams, W. E., M.D ........ Augusta, Ga.
Alexander, J. F., M.D ........ Atlanta, Ga.
Allen, Jos. Eve, M.D ........ Augusta, Ga.
Anthony, E. R., M.D ........ Griffin, Ga.
Arnold, C. D., M. D ........ El Reno, Oklahoma Ter.
Barnum, R. E. L., M.D ........ Richland, Ga.
Battey, Wm. Whatley, M.D ........ Augusta, Ga.
Bizzell, B. W., M.D ........ Atlanta, Ga.
Brown, George, M.D ........ Atlanta, Ga.
Campbell, J. L., M.D ........ Atlanta, Ga.
Champion, W. L., M.D ........ Atlanta, Ga.
Childs, J. A., M.D ........ Atlanta, Ga.
Clark, M. A., M.D, ........ Macon, Ga.
Cooper, Hunter P., M. D ........ Atlanta, Ga.
Crawford, J. M., M.D ........ Atlanta, Ga.
Davis, E. C., M.D ........ Atlanta, Ga.
Dawbarn, R. H. M., M.D ........ New York, N. Y.
Duggan, Wm. C., M.D ........ Louisville, Ky.
Ellett, E. C., M.D ........ Memphis, Tenn.
Eshner, A. A., M.D ........ Philadelphia, Pa.
Felder, L. A., M.D ........ Atlanta, Ga.
Fite, C. C., M. D ........ New York.
Foucher, A. A., M.D ........ Montreal, Canada.
Foy, Geo., M.D ........ Dublin, Ireland.
Gaston, J. McFadden, M.D ........ Atlanta, Ga.
Gillespie, William, M.D ........ Cincinnati, O.
Goggans, J. A., M.D ........ Alexander City, Ala.
Goidtsnoven, E. Van, M.D ........ Atlanta, Ga.
Goldsmith, W. S., M.D ........ Atlanta, Ga.
Gottheil, Wm. S., M.D ........ New York.
Graham, St. Joseph B., M.D ........ Savannah, Ga.
Hagan, Hugh, M.D ........ Atlanta, Ga.
Harrison, V. W., M.D ........ Richmond, Va.
Hudson, W. H., M.D ........ LaFayette, Ala.
Hutchins, M. B., M.D ........ Atlanta, Ga.
Jackson, Edw. B., M.D ........ Houston, Texas.
Kilpatrick, A. J., M.D ........ Augusta, Ga.
Kime, R. R., M.D ........ Atlanta, Ga.
Levy, E. C., M.D ........ Richmond, Va.
Ludlow, Ogden C., M.D ........ New York, N. Y.
Marcus, Herman D., M.D ........ Philadelphia, Pa.

CONTRIBUTORS—*continued.*

Mitra, N. C., A.M., M.D. ....... ....................... .........Ranchi, Bengal, India.
Noble, Geo. H., M.D............ ................ .................................Atlanta, Ga.
Olmsted, Jno. C., M.D.............................................................Atlanta, Ga.
Patterson, A. Bethune, M.D........ ......... ........................................Atlanta, Ga.
Pope, Curran, M.D................ ...................................................Louisville, Ky.
Powell, T. O., M.D........................... .................. .............Milledgeville, Ga.
Roy, Dunbar, M.D..................... ........................................ ....... Atlanta, Ga.
Slack, H. R., M.D................. ........... ................................. LaGrange, Ga.
Smith, Charles H. (Bill Arp) ................ ...... ..........................Cartersville, Ga.
Smith, S. Mac Cuen, M.D.................... ...........................Philadelphia, Pa.
Spratling, W. P., M.D..... ......... ....................... ......................Sonyea, N. Y.
Stirling, A. W., M.D .... ......................... ....... .........................Atlanta, Ga.
Stone, I. S., M.D...... ....... ............... ..................................Washington, D. C.
Terrell, Henry W., M.D............................. ..............................Greenville, Ga.
Todd, J. S., M.D...... ...................................................................Atlanta, Ga.
Upshur, Jno. N., M.D......................... ............................ ... ....Richmond, Va.
Van Marter, J. G., Jr., M.D............... ......................................Savannah, Ga.
Williams, Howard J., M.D..............................................................Macon, Ga.
Wolff, Bernard, M.D ................................................ ..... ................Atlanta, Ga.

